# Translating AI and peer feedback in sustainability-oriented STEM teacher education: pre-service mathematics teachers’ uptake, revision, and modeling task-design quality

**DOI:** 10.3389/fpsyg.2026.1922989

**Published:** 2026-10-06

**Authors:** Sejun Oh

**Affiliations:** Department of Mathematics Education, Hongik University, Seoul, Republic of Korea

**Keywords:** feedback literacy, feedback translation, generative AI, mathematical modeling, peer feedback, pre-service mathematics teacher education, sustainability-oriented STEM education, task design

## Abstract

**Introduction:**

As generative AI becomes increasingly available in teacher education, a central question is how pre-service teachers interpret, evaluate, and translate different feedback sources into coherent design decisions. This study examined how 22 South Korean pre-service mathematics teachers used AI and peer feedback while revising sustainability-oriented mathematical modeling tasks on school cafeteria food waste, a context aligned with Sustainable Development Goal 12.

**Methods:**

Using an exploratory, comparative mixed-methods sequence-comparison design embedded in an authentic teacher-education course, participants received two rounds of feedback in different sequences: AI followed by peer feedback or peer feedback followed by AI. Framed within cognitive, affective, and behavioral dimensions of feedback engagement, the analysis combined double-coded ratings of initial and final task designs, feedback-response records, revision-depth coding, validation-focused revision indicators, and post-activity reflections.

**Results:**

Participants rarely copied feedback passively: 20 selectively accepted feedback in at least one round, and 17 made at least one structural or reconceptual revision. Five participants showed a positive composite gain in rated task-design quality. Participants valued AI feedback for its specificity, new perspectives, and speed, and more often made validation-focused revisions after it, whereas they valued peer feedback for mutual communication, classroom realism, and student perspective. Descriptive comparisons showed different engagement trajectories across the sequence conditions alongside similar composite-quality changes.

**Discussion:**

Feedback translation provides an analytic perspective on the divergence between revision activity and artifact quality. Within this course context, the findings show how feedback evaluation, revision decisions, and artifact quality can diverge and suggest that teacher educators should scaffold evaluative judgment, source comparison, and post-revision coherence checking.

## 1 Introduction

Generative artificial intelligence (AI) has rapidly changed the feedback ecology of teacher education. Large language models (LLMs) can now provide immediate, detailed, and seemingly individualized comments on lesson plans, assessment items, student work, and instructional materials, including tasks designed by pre-service teachers (PSTs) (Kasneci et al., 2023; Yan et al., 2024). Recent studies suggest that AI-generated feedback can approximate human feedback on some indicators of specificity, coverage, and consistency (Banihashem et al., 2024; Steiss et al., 2024). These affordances are especially attractive in teacher education, where instructor time is limited and where PSTs need repeated opportunities to refine complex professional artifacts. However, faster and more abundant feedback does not necessarily produce deeper learning. The educational value of feedback depends not only on what is provided, but also on how learners interpret, evaluate, and act on it (Boud and Molloy, 2013; Carless and Boud, 2018). This distinction is particularly important as AI feedback becomes integrated with, rather than simply substituted for, peer feedback in teacher-education programs.

Sustainability-oriented mathematical modeling task design provides a demanding context in which to examine this issue. Mathematical modeling requires learners to formulate a real-world situation mathematically, work within a model, interpret results, and validate them in relation to the original context (Blum and Niss, 1991; Niss et al., 2007). From a models-and-modeling perspective, modeling is not merely an application of previously learned mathematics; it is a form of mathematical thinking in which representations, assumptions, data, and interpretations are developed together (Lesh and Doerr, 2003). Designing such tasks is therefore a sophisticated professional competence for PSTs. They must identify a meaningful context, formulate an open modeling question, decide what data and variables students will need, anticipate multiple possible modeling approaches, and design validation procedures that go beyond checking a numerical answer (Doerr et al., 2017; Maaß, 2010). Sustainability-oriented tasks intensify these demands because problems related to the Sustainable Development Goals (SDGs) are often open, value-laden, data-dependent, and connected to social action (Jeong and González-Gómez, 2022; United Nations, 2015). In such tasks, feedback uptake is consequential because a single suggestion can alter the realism of the context, the openness of the problem, the structure of the model, or the validity of the proposed decision.

Existing research has clarified important differences between AI and peer feedback, but much of this work remains organized by a source-comparison logic. Studies often ask whether AI-generated feedback is more accurate, detailed, efficient, or useful than peer feedback or human feedback (Banihashem et al., 2024; Steiss et al., 2024). This literature is valuable because it identifies distinctive affordances and risks. AI feedback can be fast, comprehensive, and consistent, but it can also be decontextualized, overly fluent, or insufficiently sensitive to local pedagogical goals (Bearman and Ajjawi, 2023; Kasneci et al., 2023; Yan et al., 2024). Peer feedback can be socially grounded, dialogic, and useful for developing evaluative judgment, but it can also vary in specificity, accuracy, and confidence (Liu and Carless, 2006; Nicol et al., 2014; van Popta et al., 2017). Yet source comparison leaves a central learner-side process underexplored: how PSTs judge feedback from different sources and convert it into revisions of a professional design artifact.

To address this gap, feedback can be examined through uptake, evaluative judgment, and translation. Feedback is not a completed instructional act when comments are delivered; it becomes educationally consequential only when learners make sense of it and decide what to do with it (Boud and Molloy, 2013; Carless and Boud, 2018). Feedback-literate learners are able to appreciate feedback, make judgments about quality, manage affect, and take action (Carless and Boud, 2018; Molloy et al., 2020). Similarly, evaluative judgment involves the capacity to make informed decisions about the quality of one’s own and others’ work (Tai et al., 2018). These ideas are especially relevant for PSTs, who must learn to evaluate pedagogical suggestions rather than merely comply with them. In the present study, feedback translation is used as an analytic perspective for tracing the event-level pathway through which a particular external comment is judged, accepted, adapted, rejected, or left unresolved and then connected to an observable revision and its artifact-level consequence. Along this pathway, uptake is the immediate stance taken toward a comment, evaluative judgment is the capability used to assess its quality, and feedback literacy is the broader capacity that supports both; translation links these constructs from the comment to design action and resulting task quality.

The sequence in which PSTs encounter AI and peer feedback also matters, although no order should be assumed to be superior. Feedback received earlier in a process can shape expectations, attention, and the interpretation of later feedback (Carless and Winstone, 2023; Hattie and Timperley, 2007). In combined AI-peer environments, however, sequence is better understood as an ordering context than as a simple test of which feedback source should come first. AI and peer feedback may prompt different forms of engagement. AI may foreground technical completeness, alternative formulations, or validation demands; peer feedback may foreground classroom realism, student accessibility, and the social plausibility of a task. The key question is therefore how PSTs compare, filter, and translate these sources across rounds of revision.

Feedback research also needs to distinguish revision activity from improvement. In complex design tasks, a structural change may strengthen one feature while weakening the coherence of the whole task; a reasoned rejection may preserve the designer’s intention while leaving another modeling problem unresolved; and a small textual change may substantially clarify the modeling goal. Sustainability-oriented modeling task quality therefore depends on how revisions coordinate context, data, assumptions, mathematical structure, validation, and action. In this study, the feedback translation gap refers to a mismatch between visible revision activity and final task-quality improvement. The term directs attention to how PSTs transform feedback into design decisions and to the coherence of the resulting artifact.

The purpose of this study is to examine how pre-service mathematics teachers translate AI and peer feedback into revisions of sustainability-oriented mathematical modeling tasks, and how these translation processes relate to final task quality. The study analyzes the design records of 22 PSTs who designed modeling tasks on school cafeteria food waste, a sustainability problem aligned with SDG 12. Participants received two rounds of feedback in different orders: AI followed by peer feedback or peer followed by AI feedback. The analysis integrates rated task quality before and after feedback, coded feedback-response records, revision-depth indicators, and reflective responses about AI and peer feedback. Sequence serves as an ordering context for examining feedback uptake, revision patterns, and multidimensional engagement across the two rounds.

The study is guided by three research questions:

RQ1. How do pre-service mathematics teachers selectively accept, modify, or reject AI and peer feedback when designing sustainability-oriented mathematical modeling tasks?

RQ2. What kinds and depths of task-design revisions do pre-service mathematics teachers make after AI and peer feedback, and when do these revisions improve or fail to improve final task quality?

RQ3. How do pre-service teachers describe and enact the cognitive, affective, and behavioral roles of AI and peer feedback when revising sustainability-oriented mathematical modeling tasks?

This study contributes to research on AI-supported teacher education in three ways. First, it offers an analytic perspective for tracing how PSTs judge and transform feedback from AI and peer sources. Second, it distinguishes revision depth from final task-quality improvement, thereby challenging the assumption that more visible revision necessarily indicates better learning or better design. Third, it describes how participants characterized and used the cognitive, affective, and behavioral resources associated with AI and peer feedback in sustainability-oriented mathematics teacher education. These contributions inform the design of feedback-rich teacher-education environments in which generative AI and peer interaction are not treated as competing feedback sources, but as resources that PSTs must learn to evaluate, negotiate, and translate.

## 2 Theoretical background

### 2.1 Sustainability-oriented mathematical modeling task design as professional design competence

Mathematical modeling has long been regarded as a central component of mathematics education because it connects mathematical reasoning with real-world situations, assumptions, data, and interpretation (Blum and Niss, 1991; Pollak, 1969). In modeling, learners do not simply apply a known procedure to a given problem. They must construct a representation of a situation, decide what variables matter, formulate relationships among those variables, work mathematically within the representation, and interpret the result in relation to the original situation (Niss et al., 2007). The models-and-modeling perspective further emphasizes that modeling activities involve the development and refinement of conceptual systems, not merely the production of an answer (Lesh and Doerr, 2003). For teacher education, this means that modeling-task design is itself a professional practice requiring specialized pedagogical judgment.

Designing a worthwhile modeling task is difficult because several quality dimensions must be coordinated simultaneously. A task should be grounded in a real-world context that is meaningful enough to invite inquiry, but mathematically structured enough to support modeling. It should provide or invite data that students can use productively, but it should not reduce modeling to a closed calculation. It should allow assumptions, choices, and multiple solution paths, but it should also guide students toward mathematically defensible representations. It should include validation opportunities, but validation should involve comparison with reality, sensitivity to assumptions, or evaluation of consequences rather than simple answer checking (Maaß, 2010; Niss et al., 2007). These design features are challenging for PSTs because they require both mathematical knowledge and pedagogical imagination (Borromeo Ferri, 2018; Doerr et al., 2017).

Sustainability-oriented modeling tasks make this design competence even more demanding. SDG-related problems, such as food waste, energy use, consumption, water, or transportation, often require students to coordinate quantitative reasoning with social, environmental, and ethical considerations (United Nations, 2015). In such settings, the task designer must decide how much contextual information to provide, what data are plausible, which assumptions are pedagogically useful, and how students might justify an action or decision. This aligns with multidimensional approaches to STEM education for sustainable development, which emphasize cognitive, affective, and behavioral dimensions of learning (Jeong and González-Gómez, 2022). In Korea, the 2022 revised mathematics curriculum also emphasizes mathematical modeling as a process connected to problem solving and real-world connections (Korean Ministry of Education, 2022). Thus, supporting PSTs in the design of sustainability-oriented modeling tasks is both a local teacher-education need and a broader international concern.

Feedback is an important resource for developing this competence, but it is not a simple corrective mechanism. Because modeling-task design involves trade-offs among context, openness, data, model structure, validation, and action, feedback suggestions must be interpreted in relation to the task designer’s goals. A suggestion that improves accessibility may reduce openness; a suggestion that increases realism may make the mathematical model harder to construct; a suggestion that adds data may strengthen validation but overload students. For this reason, the development of modeling-task design competence depends not only on receiving feedback, but also on learning how to evaluate feedback and translate it into coherent design decisions.

### 2.2 Feedback as uptake, evaluative judgment, and design revision

Contemporary feedback research increasingly treats feedback as a learner-centered process rather than a one-way transmission of information. In this view, feedback is not completed when comments are delivered; it becomes feedback for learning when students interpret the information, compare it with standards or goals, and take action (Boud and Molloy, 2013; Carless and Boud, 2018). This distinction is crucial in teacher education because PSTs are not only producing an artifact for assessment. They are learning how to judge the quality of pedagogical designs and how to justify changes in relation to learners, content, and context.

The concept of feedback literacy provides a useful lens for this process. Carless and Boud (2018) describe student feedback literacy as involving appreciation of feedback, making judgments, managing affect, and taking action. Molloy et al. (2020) further emphasize that feedback literacy should be understood as a learning-centered capacity that develops through practice and participation in feedback processes. These ideas shift attention from the quality of feedback alone to the learner’s capacity to use feedback productively. For PSTs, feedback literacy includes the ability to decide whether a suggestion fits the mathematical goal of the task, whether it is realistic for students, whether it strengthens or weakens validation, and whether it aligns with the intended sustainability context.

Evaluative judgment is closely related to feedback literacy. Tai et al. (2018) define evaluative judgment as the capability to make decisions about the quality of one’s own and others’ work. Peer feedback is often valued because it can develop this capability: when learners evaluate a peer’s work, they encounter alternative approaches and standards that can inform their own subsequent judgments (Nicol et al., 2014; van Popta et al., 2017). AI feedback may also support evaluative judgment when learners treat it as a resource to examine rather than an authority to obey. In both cases, the core educational process is not acceptance alone, but judgment.

This study therefore conceptualizes feedback uptake as selective and interpretive. PSTs may accept a suggestion as written, modify it to fit their own task, reject it with reasons, or leave it unresolved. These responses are not merely compliance categories. They indicate how PSTs position themselves in relation to external advice and how they exercise agency in design. Winstone et al. (2017) argue that learners’ agentic engagement with feedback includes seeking, making sense of, and using feedback. In the present context, such engagement is visible in the records of what PSTs found useful, what they found unconvincing, what they changed, and why they believed their own judgment was needed.

Revision is the behavioral trace of this interpretive process, but revision should not be equated with improvement. Revision can be surface-level, such as adding a phrase or clarifying wording; structural, such as adding data, assumptions, validation, or model elements; or reconceptual, such as reframing the central modeling question. In writing research, revision has long been treated as a process of re-seeing and reorganizing meaning rather than simply correcting errors (Fitzgerald, 1987; Sommers, 1980). A similar distinction is useful for modeling-task design. A task may be revised deeply without becoming better if the revision introduces incoherence, reduces openness, or weakens the fit between the model and the context. Conversely, a limited revision can improve quality if it clarifies the modeling goal or strengthens validation. For this reason, feedback research in complex design domains must distinguish uptake, revision depth, and final artifact quality.

### 2.3 AI-generated feedback and peer feedback as complementary resources

AI-generated feedback and peer feedback differ in the kinds of resources they make available to PSTs. AI feedback can be produced quickly, repeatedly, and in response to detailed prompts. Studies of AI-generated feedback suggest that LLMs can provide comments that are specific, extensive, and comparable to human feedback on some quality indicators (Banihashem et al., 2024; Steiss et al., 2024). In task-design contexts, these affordances may help PSTs identify missing elements, consider alternative formulations, or notice the need for more explicit validation. AI can also reduce logistical barriers to feedback by making individualized comments available when peers or instructors are not immediately available.

AI feedback also creates a professional verification task. LLMs can produce fluent but unreliable mathematical or pedagogical claims and lack direct access to local curriculum constraints, student histories, or classroom norms (Bender and Koller, 2020; Frieder et al., 2023; Kasneci et al., 2023). Because authoritative presentation can mask uncertainty, PSTs need to check AI suggestions against mathematical goals, pedagogical context, and student accessibility (Bearman and Ajjawi, 2023; Yan et al., 2024). Used in this way, AI feedback becomes a resource for design learning and evaluative judgment.

Peer feedback offers different affordances. Because peers are situated in the same course, task context, and developmental stage, their comments can reflect practical concerns that AI may miss. Peer feedback can foreground whether a task seems understandable to students, whether the context is plausible, whether the activity feels classroom-feasible, or whether the proposed question invites meaningful discussion. Research on peer feedback emphasizes its role in developing reflection, evaluative judgment, and dialogic engagement (Liu and Carless, 2006; Nicol et al., 2014; Topping, 1998; van Popta et al., 2017). In teacher education, these affordances are especially relevant because PSTs need to learn how others may interpret their instructional designs.

Peer and AI feedback provide complementary resources whose value depends on how PSTs use them. Peer comments can contribute contextual realism, student perspective, and social negotiation, while also varying in specificity and confidence. AI feedback can contribute technical articulation, alternative generation, and validation prompts, while requiring contextual and mathematical verification. The theoretical issue is how PSTs compare and translate these resources into design decisions.

### 2.4 Feedback sequencing as an ordering context

Feedback often unfolds over time, and earlier feedback may influence how later feedback is interpreted. Hattie and Timperley (2007) describe feedback as involving questions about goals, current performance, and next steps, and Carless and Winstone (2023) emphasize that feedback practices are shaped by the interplay between teacher and student feedback literacy. In multi-source feedback environments, sequence may affect attention, expectations, and comparison. A first feedback source may establish what the learner sees as important, and a second source may confirm, complicate, or challenge that initial frame. This makes sequence theoretically relevant.

In AI-peer feedback environments, sequence can organize the evaluative resources available at different moments of revision. AI-first feedback may provide technical language that helps PSTs notice task-design elements before peer discussion, whereas peer-first feedback may establish social and contextual grounding before PSTs evaluate AI suggestions. The educational significance of either order lies in how learners compare and translate the feedback they receive across rounds.

The present study therefore treats feedback sequence as an ordering context for comparing how PSTs encounter two feedback sources, record their uptake, and revise across rounds. This framing aligns sequence with the study’s focus on feedback translation and makes patterns of acceptance, modification, rejection, and revision the central objects of comparison.

### 2.5 A multidimensional framework for feedback translation

Sustainability-oriented STEM education requires attention to cognitive, affective, and behavioral dimensions of learning (Jeong and González-Gómez, 2022). This multidimensional framing is well suited to the study of AI and peer feedback because feedback engagement is not only cognitive. PSTs must interpret feedback and reason about task quality; they must also manage trust, uncertainty, confidence, and concern about the feedback source; and they must decide whether and how to revise the task. A multidimensional framework therefore helps explain why the same feedback comment may lead to different forms of action.

In the present study, the cognitive dimension concerns how PSTs interpret feedback in relation to mathematical modeling task quality. This includes recognizing whether a suggestion concerns the real-world context, the openness of the question, data and assumptions, mathematical structure, validation, or decision-making. The affective dimension concerns how PSTs perceive the credibility, usefulness, limitations, and risks of AI and peer feedback. This includes trust in AI specificity, caution about AI errors, appreciation of peer realism, or frustration with vague peer comments. The behavioral dimension concerns the observable actions PSTs take, including accepting, modifying, rejecting, or leaving feedback unused, and making surface, structural, or reconceptual revisions.

Feedback translation connects these dimensions. A PST may cognitively recognize that a suggestion is relevant, affectively distrust the source, and behaviorally modify the suggestion rather than accept it directly. Another PST may trust the source but fail to integrate the suggestion coherently into the task. A third may reject a suggestion for defensible contextual reasons but still leave a deeper modeling weakness unresolved. These possibilities explain why feedback provision, feedback uptake, revision depth, and final task quality must be analytically separated.

This separation motivates the notion of a feedback translation gap: PSTs may visibly engage with feedback and make substantial revisions without improving the rated quality of the final modeling task. The notion challenges a linear model in which more feedback leads to more revision and more revision leads to better work, and it organizes case-level evidence about where feedback engagement, revision activity, and rated quality diverged.

The framework developed here positions AI and peer feedback as resources within a feedback-rich design environment. PSTs must learn not only to receive feedback, but also to translate feedback into defensible professional action. This perspective directly informs the study’s three research questions about selective uptake, revision depth and quality translation, and the cognitive, affective, and behavioral functions of AI and peer feedback in sustainability-oriented mathematical modeling task design.

## 3 Materials and methods

### 3.1 Research design

This study used an exploratory, comparative mixed-methods sequence-comparison design embedded in an authentic teacher-education course. The two ordering conditions were AI-to-peer, in which PSTs received AI feedback in the first round and peer feedback in the second, and peer-to-AI, in which the order was reversed. Group-level ordering defined the comparative context, and the analysis centered on within-course patterns of feedback uptake, revision depth, and rated task quality across the two conditions. Sequence was implemented at the group level rather than through individual random assignment or counterbalancing. Because each second round followed exposure to a different first source and an intervening revision, the observed patterns cannot separate ordering from group composition, task maturity, or carryover. The design therefore supports descriptive within-course comparisons rather than causal estimates of source or order effects.

The study combined product-oriented and process-oriented data. Product-oriented data consisted of double-coded ratings of the initial and final modeling tasks. Process-oriented data consisted of PSTs’ written feedback-response records, revision notes, and reflections on AI and peer feedback. This combination made it possible to distinguish four related but analytically separate constructs: task quality, feedback uptake, revision depth, and perceived feedback function.

Figure 1 summarizes the two feedback-sequence workflows and links each activity stage to the product, process, and reflective evidence analyzed.

**Figure 1:**
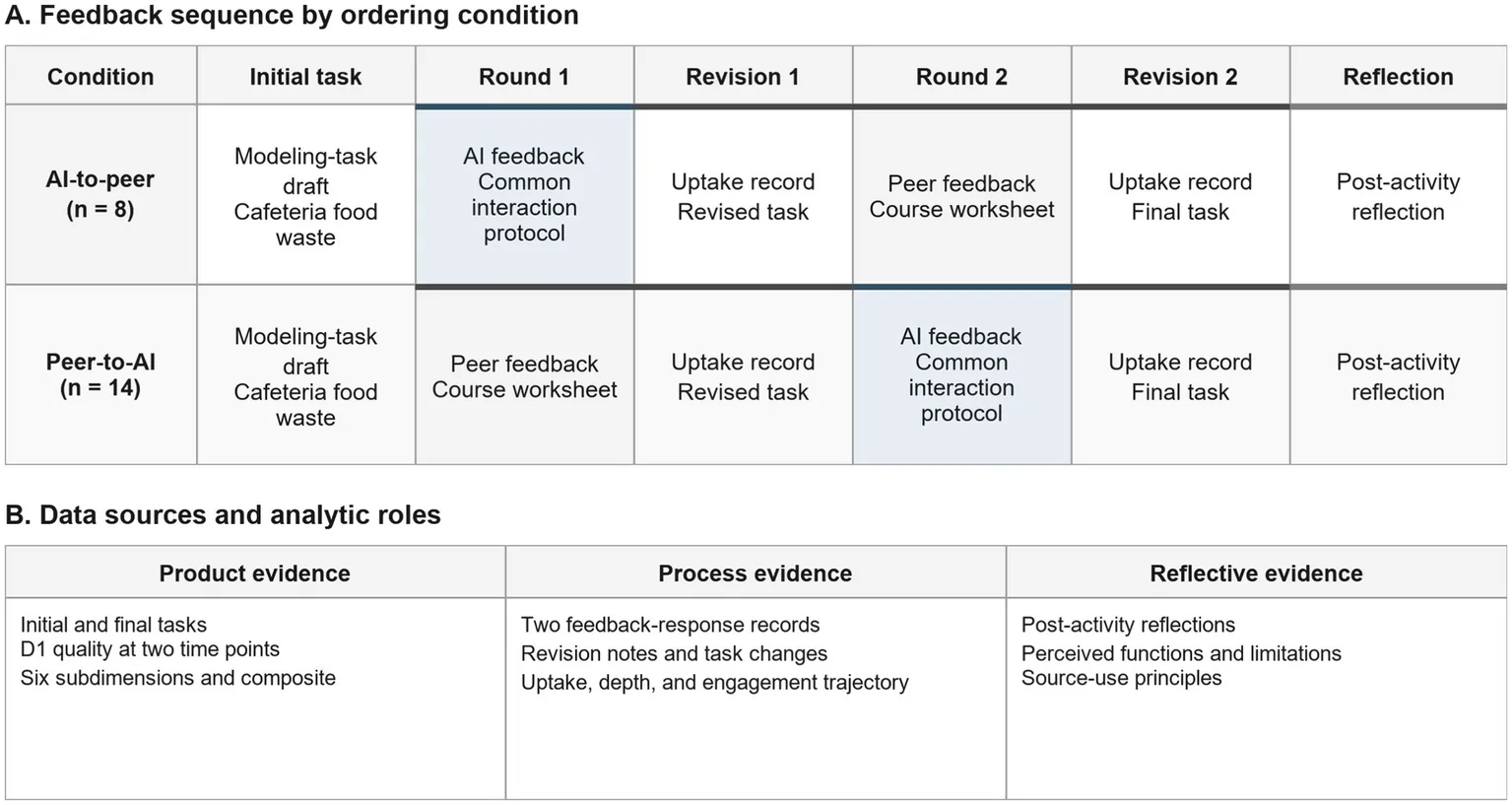
Study workflow and analytic alignment. **(A)** Feedback sequence by ordering condition: feedback order was assigned at the group level in an authentic course; all 22 PSTs completed an initial task, two feedback-response-revision rounds, a final task, and a post-activity reflection. **(B)** Data sources and analytic roles.

### 3.2 Participants and instructional context

Participants were 22 pre-service mathematics teachers enrolled in a required undergraduate course on logic and essay writing in mathematics education at a four-year university in South Korea during the spring 2026 semester. The course forms part of a mathematics-teacher preparation program and includes instruction on mathematical task design, mathematical modeling, argumentation, and written justification.

The analytic sample was defined before analysis as the set of PSTs for whom complete paired data were available for the present research questions. Inclusion required (a) assignment to one of the two feedback-sequence conditions, (b) completion of the initial modeling-task draft, both feedback rounds, and the final revised task, and (c) external ratings for both the initial and final task designs. Twenty-two PSTs met these criteria. Eight PSTs were in the AI-to-peer condition and 14 were in the peer-to-AI condition. All descriptive summaries, condition comparisons, and cross-case analyses in this article use this fixed analytic sample of 22 PSTs.

Feedback sequence was assigned at the group level to preserve the course’s peer-review workflow, producing condition sizes of eight and 14.

### 3.3 Modeling task and feedback procedure

The study was embedded in a seven-week instructional unit within a 15-week course. The unit introduced the 2022 revised Korean mathematics curriculum, mathematical modeling, sustainability-oriented task design, and the use of AI and peer feedback for revising mathematical tasks. The culminating activity required PSTs to design a mathematical modeling task on reducing school cafeteria food waste, a context aligned with Sustainable Development Goal 12, Responsible Consumption and Production.

PSTs completed a structured modeling-task design worksheet. The worksheet guided them through an initial exploration of the problem context, identification of relevant variables and data, formulation of modeling questions, and construction of a complete student-facing modeling task. The initial task draft included the following components: task title, target grade level, key mathematical concepts, situation prompt, modeling question, data, assumptions, model or representation, validation procedure, and decision or action component.

After completing the initial draft, each PST received two rounds of feedback. In the AI-to-peer condition, the first round was generated by AI and the second was provided by a peer; the order was reversed in the peer-to-AI condition. For every AI round, each PST used the same course-specific custom GPT in ChatGPT.^1^ At the time of data collection, the custom GPT ran on the latest GPT-5-series model available in ChatGPT and applied a common three-stage interaction protocol: identity verification, question-and-answer interaction, and automated logging. The instruction did not prescribe a fixed domain-specific feedback template; the substantive AI response therefore varied with the PST’s original question and the model-generated output. The custom GPT interface and interaction protocol were held constant across participants and sequence conditions. Peer feedback was exchanged in writing during class through the course activity worksheet.

After each feedback round, PSTs recorded their response to the feedback. For the AI rounds, the custom GPT automatically logged the student’s name, student identification number, original question, and generated response in a Google Sheet. Students transferred the question-and-response exchanges verbatim into their submitted feedback-response reports. The transferred entries were cross-checked against the source logs to verify verbatim correspondence and the completeness of transfer. The submitted reports constituted the primary analytic artifacts; the source logs served as verification records and were not counted as an independent second set of observations. PSTs also indicated which feedback points they found useful or unconvincing, what they intended to revise, how they accepted or rejected the feedback, and why their own judgment was needed. They revised the task before receiving the next feedback source and, after the second round, submitted a final revised task and completed written reflections on the usefulness, limitations, and appropriate use of AI and peer feedback.

### 3.4 Data sources

The analysis drew on four data sources, each aligned with one or more research questions.

First, the initial and final modeling tasks were used to assess task-design quality. The initial task was the complete draft submitted before either feedback round, and the final task was the version submitted after both feedback rounds. These two artifacts provided the basis for pre-post Domain 1 ratings.

Second, submitted feedback-response reports from the first and second rounds were used to analyze feedback uptake and revision behavior. For the AI rounds, these reports contained verbatim question-and-response exchanges whose transfer had been checked against the corresponding Google Sheets source records. The reports also included checkbox responses and open-ended explanations about what PSTs accepted, modified, rejected, or left unresolved.

Third, revision notes and final task changes were used to classify the depth and type of task-design revision. These data made it possible to distinguish surface edits from structural and reconceptual changes, and to examine whether those revisions corresponded to improvements in specific dimensions of task quality.

Fourth, post-activity reflections were used to analyze PSTs’ perceptions of the strengths, limitations, and appropriate use of AI and peer feedback. These reflections supported the analysis of cognitive, affective, and behavioral functions of the two feedback sources.

All artifacts were anonymized before external rating. Student names, student identification numbers, and group-member names were removed from the rater package. Feedback-source indicators were retained where necessary because they were required for coding feedback sequence and uptake.

### 3.5 Measures and coding scheme

The coding scheme was adapted from the study codebook used for external rating and focused on four domains relevant to the present research questions: task-design quality, feedback engagement, perceived feedback functions, and validation focus.

#### 3.5.1 Domain 1: task-design quality

Domain 1 (hereafter D1) captured the quality of the modeling task before and after feedback. Each initial and final task was scored on six subdimensions, each using a 0–2 anchored scale: (a) life-context connection, (b) openness, (c) quantity, assumptions, data, and constraints, (d) modeling requirement, (e) validation or reality interpretation, and (f) discussion or collaboration. Composite D1 scores ranged from 0 to 12 and were calculated as the sum of the six subdimension scores. Equal weighting treats the six features as co-required elements of task design and avoids privileging one feature without an empirical basis. The composite is interpreted as an indicator of the breadth and joint representation of design features rather than as a latent interval measure; subdimension changes are therefore reported alongside the total so that offsetting gains and declines remain visible.

The six subdimensions correspond to central design features of sustainability-oriented mathematical modeling tasks. Life-context connection captures whether the task is anchored in a meaningful school, community, or stakeholder context. Openness captures whether multiple approaches, models, criteria, or decisions are possible. Quantity, assumptions, data, and constraints capture whether the task provides or invites mathematically usable information. Modeling requirement captures whether students must construct or use a mathematical model rather than perform direct computation. Validation captures whether students are asked to check, compare, or interpret model outputs in relation to reality. Discussion or collaboration captures whether the task invites communication, negotiation, or decision-making among students.

#### 3.5.2 Domain 2: feedback engagement and revision depth

Domain 2 captured how PSTs responded to each feedback round. Feedback uptake was coded for each round using five possible categories: accept-as-is, selective acceptance, modified acceptance, rejection, and hold or unresolved. Accept-as-is indicated that the PST reported using the feedback without substantive adaptation. Selective acceptance indicated that the PST accepted some elements while rejecting or qualifying others. Modified acceptance indicated that the PST used the feedback but transformed it to fit the task, context, or instructional intention. Rejection indicated that the PST explicitly declined a suggestion and provided a reason. Hold or unresolved indicated that the PST did not make a clear decision about the feedback.

Revision depth was coded separately for each feedback round on a 0–3 scale: 0 = no visible revision, 1 = surface revision, 2 = structural revision, and 3 = reconceptual revision. Surface revision referred to wording changes, minor additions, or clarification that did not substantially alter the task design. Structural revision referred to changes that added or reorganized task components such as data, assumptions, modeling procedures, validation steps, or student decision structures. Reconceptual revision referred to changes that reframed the central modeling question, the task logic, or the intended modeling activity.

Domain 2 also included a critical-engagement trajectory based on the strength and consistency of PSTs’ written reasoning across the two rounds. These trajectories were classified as asymmetric, improving, medium-consistent, or strong-consistent. This classification was used to examine whether PSTs’ engagement with feedback remained stable, became more explicit across rounds, or differed depending on the feedback source.

#### 3.5.3 Domain 3: perceived functions of AI and peer feedback

Domain 3 captured PSTs’ perceptions of AI and peer feedback in their post-activity reflections. Reflections were coded thematically for perceived strengths, perceived limitations, and stated principles for appropriate use. AI-related themes included specificity, speed, provision of alternative perspectives, objectivity or standardization, risk of error, lack of local context, and the need for verification. Peer-related themes included classroom realism, student perspective, mutual communication, contextual fit, slower process, possible bias, and variable specificity.

These themes were then interpreted within a cognitive, affective, and behavioral framework. Cognitive functions referred to how a feedback source supported task interpretation, model clarification, validation reasoning, or design alternatives. Affective functions referred to trust, confidence, caution, perceived usefulness, and vigilance toward the feedback source. Behavioral functions referred to how feedback prompted concrete revision actions, verification, discussion, or selective use.

#### 3.5.4 Domain 4: validation focus

Domain 4 captured validation-focused behavior across the design and revision process. Validation was coded both as a Domain 1 quality subdimension and as a cross-cutting behavioral focus. Source-specific comparisons used the round-level indicator of whether a feedback round was followed by a validation-focused revision.

### 3.6 Coding procedure and trustworthiness

All 22 analytic cases were independently coded by the author and by an external rater with training in mathematics education. The external rater was not involved in the study design, classroom implementation, or initial coding. The anonymized rating package included the codebook, rating spreadsheet, calibration materials, and student artifacts. The rater did not receive the author’s codes or the condition list. Feedback-source indicators were retained because they were necessary for coding uptake and sequence, so the order of AI and peer feedback remained inferable from the worksheet records.

Coding proceeded in two stages. First, each rater coded the artifacts independently according to the codebook anchors. Second, the two sets of codes were compared, and disagreements were resolved through evidence-based consensus discussion. Before consensus, agreement was lower for several D1 subdimensions, mainly because the raters interpreted the discussion/collaboration anchor (subdimension f) differently; five borderline validation cases (subdimension e) in the peer-to-AI condition also required adjudication. Two further questions concerned whether a validation or interpretation check was visibly recorded in an artifact and whether qualitatively different responses across rounds warranted an asymmetric trajectory label when the round-level strength ratings were the same. The coders resolved these questions by returning to the worksheet evidence and applying the written anchor and trajectory rules. All analyses reported in this article use the resulting consensus ratings.

Several steps supported trustworthiness. The codebook specified construct definitions, rating anchors, decision rules, and examples for each domain, and the external rater completed calibration cases before coding the main analytic cases. The full analytic sample was double-coded. Interpretations of feedback uptake and revision were triangulated across checkbox responses, written rationales, actual task revisions, and post-activity reflections.

### 3.7 Data analysis

The analysis was organized by research question and emphasized descriptive statistics, cross-case pattern analysis, and qualitative interpretation of feedback translation. Means, standard deviations, ranges, frequencies, and percentages were reported where appropriate. Because ordering was assigned at the group level and the condition sizes were small and unequal, no inferential test of sequence effects was used. Condition summaries describe patterns and generate hypotheses rather than establish equivalence or the absence of an effect.

For RQ1, feedback uptake was analyzed at both the feedback-episode level and the participant level. At the episode level, each AI or peer feedback event was classified by uptake category and revision depth. At the participant level, the analysis examined whether each PST showed selective acceptance, modified acceptance, rejection, structural or reconceptual revision, and consistent critical engagement across the two rounds. Patterns were summarized by feedback sequence and by feedback source.

For RQ2, task-design revisions were analyzed by linking revision-depth codes with pre-post changes in Domain 1 scores. Composite Domain 1 scores and subdimension scores were compared between the initial and final tasks. Cases were classified as improved, unchanged, or declined based on composite Domain 1 change, and positive subdimension changes were used to identify which aspects of task design improved. These analyses were used to examine the translation gap between visible revision activity and final task-quality gains.

For RQ3, post-activity reflections were analyzed thematically to identify the cognitive, affective, and behavioral roles participants attributed to AI and peer feedback. AI and peer themes were first coded separately and then compared across sources. Mixed-methods integration occurred at the case level through a matrix linking uptake codes, revision depth, Domain 1 change, Domain 4 validation indicators, and reflection themes. This matrix supported cross-case comparison and the selection of representative cases, including cases of deep revision without a D1 gain, to examine convergence and divergence between revision activity and final quality.

### 3.8 Ethics

The study was conducted in accordance with institutional research ethics policies and was reviewed and approved by the Hongik University Institutional Bioethics Committee (IRB document no. 7002340-202504-HR-002-01; approval date: April 24, 2025). Students provided written informed consent for the use of their de-identified worksheet responses, feedback records, task artifacts, and reflection data for research purposes. Students were informed that participation or non-participation in the research would not affect their course grade. All research data were anonymized before analysis and before external rating. The author retained the only link between student identities and anonymized research codes, and this link was not shared with the external rater.

## 4 Results

The analytic dataset comprised 22 PSTs with double-coded initial and final task designs: eight in the AI-to-peer condition and 14 in the peer-to-AI condition. Results are organized around within-course patterns of feedback uptake, revision activity, and multidimensional engagement across the two ordering conditions.

### 4.1 Task-design quality across the feedback cycle

The mean D1 composite changed from 7.12 to 6.88 in the AI-to-peer condition and from 6.79 to 6.50 in the peer-to-AI condition. Composite scores improved for five PSTs, were unchanged for ten, and declined for seven. Observed mean changes were similar in the two conditions (−0.25 and −0.29). The quality results therefore locate the central analytic issue in how revision activity translated into final task quality (Table 1).

**Table 1:** Task-design quality before and after feedback by sequence condition.

Outcome	AI-to-peer (*n* = 8)	Peer-to-AI (*n* = 14)	Overall (*N* = 22)
D1 initial composite	M = 7.12, SD = 2.30; range 4–12	M = 6.79, SD = 1.19; range 5–9	M = 6.91, SD = 1.63 range 4–12
D1 final composite	M = 6.88, SD = 1.55; range 4–9	M = 6.50, SD = 1.74; range 3–10	M = 6.64, SD = 1.65 range 3–10
Composite change	M = −0.25; improved 2/8 (25.0%)	M = −0.29; improved 3/14 (21.4%)	M = −0.27; improved 5/22 (22.7%)
Change distribution	Improved 2/8 (25.0%); unchanged 4/8 (50.0%); declined 2/8 (25.0%)	Improved 3/14 (21.4%); unchanged 6/14 (42.9%); declined 5/14 (35.7%)	Improved 5/22 (22.7%); unchanged 10/22 (45.5%); declined 7/22 (31.8%)

D1 composites were calculated from the six consensus-coded subdimensions so that pre- and post-feedback scores were on the same 0–12 scale.

### 4.2 Selective feedback uptake

RQ1 asked how PSTs selectively accepted, modified, or rejected AI and peer feedback. The dominant pattern was not passive acceptance. Across the analytic sample, selective acceptance was observed for 32 of the 44 feedback episodes, and 20 of the 22 PSTs showed selective acceptance in at least one round. Modified acceptance was less frequent, and explicit rejection was observed only twice, both in the peer-to-AI condition. These results indicate that PSTs generally treated feedback as material to be filtered and adapted rather than as instructions to be copied (Table 2).

**Table 2:** Feedback uptake codes by sequence condition and feedback source.

Feedback uptake code	AI-to-peer condition (16 episodes)	Peer-to-AI condition (28 episodes)	By source across conditions
Accept-as-is	2/16 (12.5%)	1/28 (3.6%)	AI: 2/22 (9.1%); Peer: 1/22 (4.5%)
Selective acceptance	10/16 (62.5%)	22/28 (78.6%)	AI: 16/22 (72.7%); Peer: 16/22 (72.7%)
Modified acceptance	4/16 (25.0%)	3/28 (10.7%)	AI: 3/22 (13.6%); Peer: 4/22 (18.2%)
Rejection	0/16 (0.0%)	2/28 (7.1%)	AI: 1/22 (4.5%); Peer: 1/22 (4.5%)
Participant-level selective uptake	7/8 (87.5%)	13/14 (92.9%)	20/22 PSTs (90.9%)

No feedback episode was coded as hold or unresolved.

The critical-engagement trajectories further clarified how uptake unfolded across the two rounds. Strong-consistent engagement appeared in two AI-to-peer cases and one peer-to-AI case, whereas improving engagement appeared more frequently in the peer-to-AI condition. This suggests that the sequence conditions differed less in the sheer presence of criticality than in the trajectory of critical engagement across rounds (Table 3).

**Table 3:** Participant-level critical-engagement trajectories across the two feedback rounds.

Trajectory pattern	AI-to-peer (*n* = 8)	Peer-to-AI (*n* = 14)	Interpretation
Asymmetric	2/8 (25.0%)	2/14 (14.3%)	One round showed stronger critical engagement than the other, without a simple improvement pattern.
Improving	1/8 (12.5%)	6/14 (42.9%)	The second-round rationale was stronger than the first-round rationale.
Medium-consistent	3/8 (37.5%)	5/14 (35.7%)	Both rounds showed moderate, reasoned engagement.
Strong-consistent	2/8 (25.0%)	1/14 (7.1%)	Both rounds showed sustained, high-level critical engagement.

### 4.3 The translation gap between deep revision and task quality

RQ2 examined what kinds and depths of task-design revisions PSTs made after feedback, and whether these revisions improved final task quality. Revision activity was widespread. Seventeen of the 22 PSTs made at least one structural or reconceptual revision, and nine did so in both feedback rounds. However, only five PSTs showed a positive D1 gain. All five quality-gain cases involved at least one structural or reconceptual revision, but most deep revisions did not translate into a higher D1 composite. This pattern is central to the results: feedback uptake and revision depth were empirically visible, but they were not equivalent to final task-quality improvement (Table 4).

**Table 4:** Revision depth and task-quality translation.

Revision indicator	AI-to-peer (*n* = 8)	Peer-to-AI (*n* = 14)	Overall
At least one structural or reconceptual revision	6/8 (75.0%)	11/14 (78.6%)	17/22 (77.3%)
Structural or reconceptual revision in both rounds	2/8 (25.0%)	7/14 (50.0%)	9/22 (40.9%)
Positive D1 gain	2/8 (25.0%)	3/14 (21.4%)	5/22 (22.7%)
Deep revision with positive D1 gain	2/6 (33.3%)	3/11 (27.3%)	5/17 (29.4%)
Deep revision without positive D1 gain	4/6 (66.7%)	8/11 (72.7%)	12/17 (70.6%)

Stability was the most common subdimension outcome. Life-context connection improved in four cases and remained unchanged in 18, whereas quantity/data showed the largest number of declines (7/22). Improvements in openness, modeling, validation, and discussion each appeared in two or three cases. Reporting improved, unchanged, and declined scores together clarifies how local gains and losses combined into the composite translation pattern (Table 5).

**Table 5:** Counts of improved, unchanged, and declined subdimension scores from initial to final task design.

D1 subdimension	AI-to-peer (*n* = 8; I/U/D)	Peer-to-AI (*n* = 14; I/U/D)	Overall (*N* = 22; I/U/D)
Life-context connection	1/7/0	3/11/0	4/18/0
Openness	0/6/2	2/10/2	2/16/4
Quantity/data	2/5/1	1/7/6	3/12/7
Modeling	1/7/0	2/10/2	3/17/2
Validation	1/5/2	1/11/2	2/16/4
Discussion	1/5/2	2/10/2	3/15/4

Figure 2 presents three English-language case pathways showing how PSTs interpreted, modified, or rejected AI and peer feedback and how those decisions related to final task quality.

**Figure 2:**
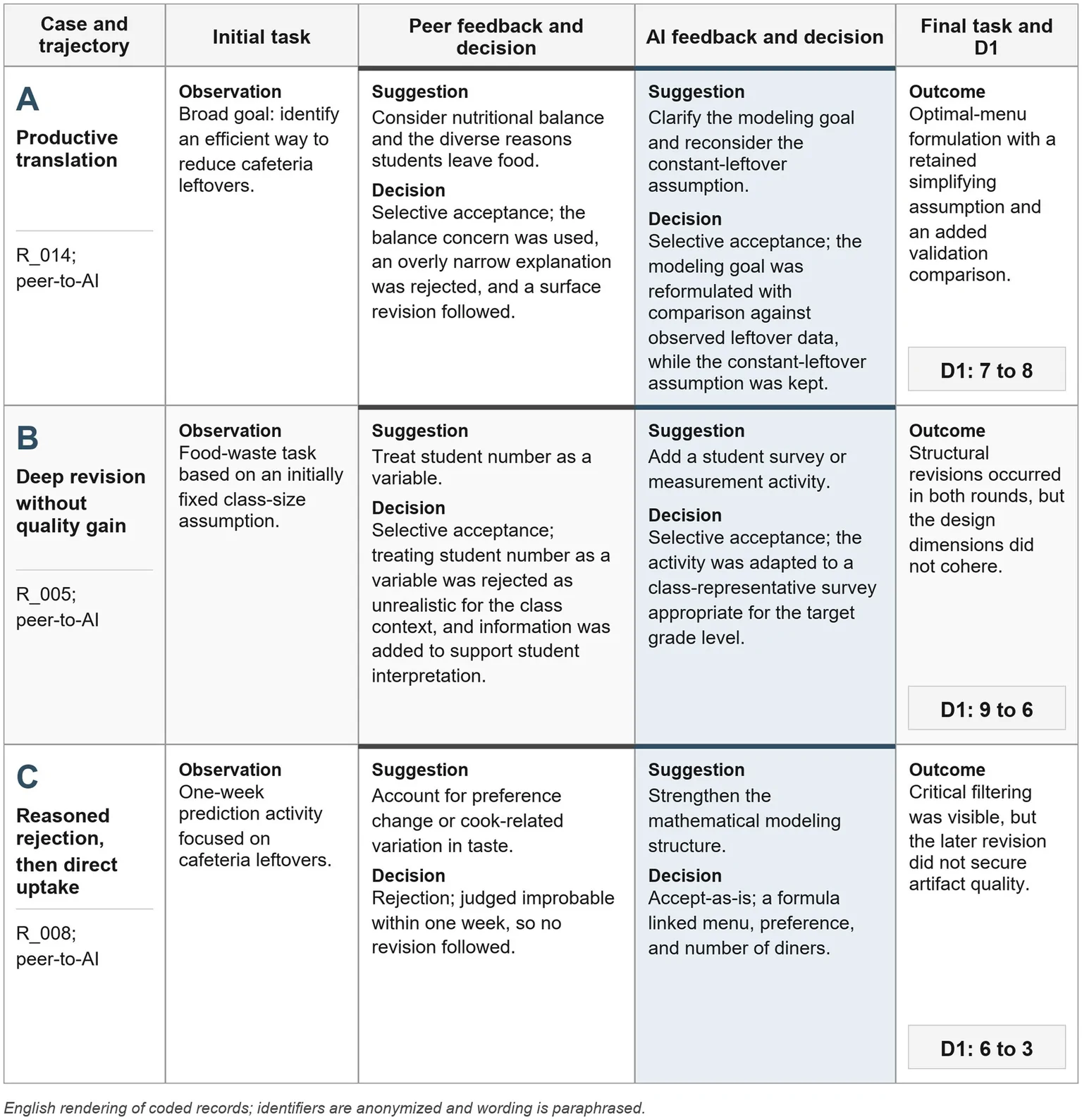
Representative pathways of feedback translation. Panel **(A)** shows productive translation across peer and AI feedback; Panel **(B)** shows deep revision without a D1 gain; and Panel **(C)** shows reasoned rejection followed by direct AI uptake. All labels and case evidence are presented in English; wording paraphrases the recorded feedback and revision entries. D1 values are consensus ratings before and after revision.

### 4.4 Participant-described roles of AI and peer feedback

RQ3 examined how PSTs described and enacted the cognitive, affective, and behavioral roles of AI and peer feedback when revising sustainability-oriented modeling tasks. Selective acceptance occurred in 16 of 22 episodes for each source. AI feedback was followed by slightly deeper revision (M = 1.68 vs. 1.45) and more validation-focused revision (11 vs. 8 of 22 episodes). In their reflections, participants most often valued AI feedback for its specificity (9), new or diverse perspectives (7), and speed (6), and peer feedback for mutual communication (8), realism and context (7), and student perspective (7) (Table 6).

**Table 6:** Participant-described and coded roles of AI and peer feedback.

Domain	AI feedback	Peer feedback	Result pattern
Behavioral uptake	Selective acceptance: 16/22 (72.7%); modified acceptance: 3/22 (13.6%); rejection: 1/22 (4.5%)	Selective acceptance: 16/22 (72.7%); modified acceptance: 4/22 (18.2%); rejection: 1/22 (4.5%)	Both sources were filtered rather than copied.
Revision depth	M = 1.68; structural/reconceptual episodes: 15/22 (68.2%)	M = 1.45; structural/reconceptual episodes: 11/22 (50.0%)	AI feedback showed somewhat deeper coded revision episodes.
Validation-oriented revision	11/22 (50.0%)	8/22 (36.4%)	Validation was more often revised after AI feedback, although final D1 validation did not uniformly increase.
Perceived strengths	specific feedback: 9; speed: 6; new/diverse perspectives: 7; objectivity: 1	realistic/contextual: 7; student perspective: 7; mutual communication: 8; new angle: 4	AI was framed as technically informative; peer feedback as pedagogically contextual.
Perceived limitations	medium vigilance/context limits: 11; high vigilance/error risk: 9; low creativity/communication limits: 2	slower than AI: 7; bias: 5; AI-comparative inferiority: 5; less specific: 4; limited scope: 3; low creativity: 3	Both sources required critical use, but the perceived risks differed.
Cross-source use principles	Not source-separated	Not source-separated	Cross-source reflection codes: verification 9; critical use 9; integration 3; auxiliary role 3; independence 2. Verification and critical use predominated.

Affective themes are reported as coded theme mentions, and cells could contain more than one theme. Use-principle themes were coded once per participant across both feedback sources and are therefore reported as cross-source reflection codes.

### 4.5 From feedback exposure to design transformation

The results across RQ1–RQ3 converge on a feedback-translation pattern. PSTs frequently engaged in selective uptake, many produced structural or reconceptual revisions, and their records associated AI and peer feedback with differently emphasized roles. Nevertheless, these revision actions did not automatically yield higher final D1 scores. Figure 3 summarizes this empirical pattern by separating feedback encounter, uptake stance, revision action, and artifact quality.

**Figure 3:**
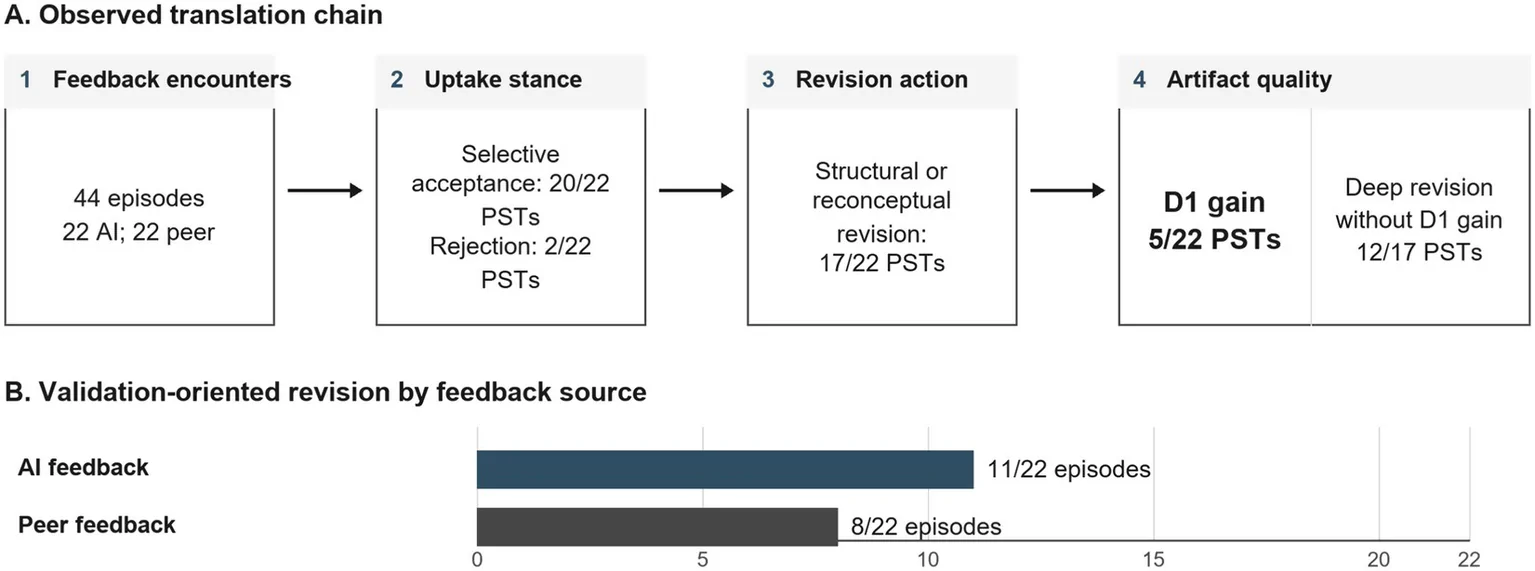
Feedback translation pattern linking uptake, revision activity, and final task-quality change. **(A)** Observed translation chain. **(B)** Validation-oriented revision by feedback source.

In sustainability-oriented mathematical modeling task design, this pattern draws attention to the coordination required after a feedback suggestion is accepted. The records show how PSTs interpreted comments and made substantive changes, while the final ratings indicate where those changes fell short of higher task quality. The relationship between these decisions and the coherence of the revised artifact is the focus of the following discussion.

## 5 Discussion

This study examined how PSTs translated AI and peer feedback into revisions of sustainability-oriented mathematical modeling tasks. The results reveal active engagement alongside uneven quality translation: 20 of the 22 PSTs selectively accepted feedback in at least one round, 17 made at least one structural or reconceptual revision, and five showed a positive D1 composite gain. This divergence is examined through feedback translation as an analytic perspective linking observed evaluative decisions, revision activity, and final task quality.

This pattern is relevant to both feedback theory and mathematics teacher education. In sustainability-oriented mathematical modeling, PSTs must interpret feedback, reconcile it with instructional intentions, and integrate it into coherent task-design decisions. This view is consistent with feedback literacy and sustainable feedback perspectives, which emphasize learners’ capacity to make evaluative judgments, act on feedback, and regulate future work (Carless and Boud, 2018; Molloy et al., 2020; Winstone et al., 2017). The contribution of the present study is to apply this analytic perspective to event-level links among feedback, design choices, and task quality in one sustainability-oriented mathematics context.

### 5.1 Feedback translation and evaluative judgment

RQ1 showed that PSTs treated both AI and peer feedback as material for professional judgment. Selective acceptance appeared in 7 of 8 AI-to-peer cases and 13 of 14 peer-to-AI cases, and it was equally common for AI and peer feedback at the source level. PSTs adopted specific suggestions, modified others to fit the task context, and rejected recommendations that conflicted with pedagogical intentions. The active work of comparison and adaptation, rather than the identity of the feedback source alone, defined feedback uptake.

This selective pattern suggests that critical engagement should be understood as a design practice, not only as a reflective attitude. In several cases, PSTs accepted feedback only after translating it into a more contextually appropriate form. In other cases, they rejected suggestions because the proposed change would make the task unrealistic, misaligned with student needs, or inconsistent with the intended modeling activity. Such decisions show that feedback literacy in task design involves more than recognizing whether a comment is “good” or “useful.” It also involves deciding how a comment should enter the design, what should be preserved, and what trade-offs a revision might create.

This interpretation is consistent with feedback literacy and evaluative judgment research, which positions learners as active judges of quality rather than as recipients of corrective information (Carless and Boud, 2018; Tai et al., 2018; Winstone et al., 2017). The present study extends that line of work by showing how such judgment becomes visible in concrete design choices about data, openness, validation, and classroom feasibility.

Following these decisions through to the revised artifact distinguishes the reasoning expressed by PSTs from the quality of the changes they made. A well-justified decision to accept or adapt a comment still leaves the practical work of integrating that change into the task.

### 5.2 The translation gap between revision effort and modeling quality

RQ2 identified a translation gap between revision depth and artifact quality. Seventeen of the 22 PSTs made at least one structural or reconceptual revision, while five showed a positive D1 composite gain, ten were unchanged, and seven declined. Visible revision effort and final task-design quality therefore represent related but distinct outcomes.

Revision research has similarly cautioned against equating revision with simple correction or guaranteed improvement (Fitzgerald, 1987; Sommers, 1980). In the present study, this distinction was especially important because mathematical modeling tasks are complex design objects whose quality depends on the coordination of context, assumptions, data, mathematical structure, validation, and discussion (Blum and Niss, 1991; Borromeo Ferri, 2018; Maaß, 2010).

There are several plausible reasons for this translation gap. First, sustainability-oriented modeling tasks are multidimensional design objects. A revision that strengthens one dimension can weaken another. For example, adding a data constraint may make the task more mathematically usable while reducing openness; specifying a validation procedure may improve realism while making the task more procedural; adding contextual detail may improve authenticity while obscuring the central modeling question. Because D1 aggregates six interdependent subdimensions, a PST can make a substantial revision without producing a net composite gain.

Second, novice task designers may recognize a legitimate problem in their draft without yet knowing how to resolve it coherently. Feedback can help PSTs notice missing data, weak validation, insufficient openness, or a vague modeling goal. However, noticing a design problem is not the same as producing a high-quality solution. Some PSTs responded to feedback by adding components to the task, but the added components did not always integrate with the modeling structure. This is especially relevant to validation. AI feedback prompted validation-focused revisions in 11 of 22 AI episodes, and peer feedback did so in 8 of 22 peer episodes, but validation gains in the final D1 ratings remained limited. The difficulty was not simply whether PSTs mentioned validation, but whether validation became a meaningful part of the modeling activity.

Third, written rationales captured within-activity evaluative reasoning that the final artifact alone could not represent. Many PSTs evaluated feedback, justified acceptance or rejection, and reflected on source credibility. These records document within-activity evaluative reasoning, while D1 scores describe the resulting artifact. Separating these outcomes keeps the quality of a justification analytically distinct from the quality of the revised task.

### 5.3 AI and peer feedback as design resources

RQ3 showed that participants associated AI and peer feedback with overlapping but differently emphasized cognitive, affective, and behavioral roles: both sources were filtered in similar ways, but AI feedback more often prompted validation-focused revision, whereas peer feedback was valued for its communicative and classroom-oriented qualities.

In the coded records, participants often used AI feedback as a cognitive resource for making task structure more explicit. Recorded suggestions addressed missing components, unclear modeling goals, data constraints, validation procedures, or alternative formulations. Participants also noted that some AI suggestions were too generic, insufficiently sensitive to classroom constraints, or misaligned with the intended task. PSTs therefore assessed AI suggestions against the demands of their own tasks.

Participants described peer feedback in relation to the perspective of a potential student, classroom peer, or future teacher. Their records linked peer comments with practical feasibility, clarity for learners, local context, and whether the task would make sense in an actual classroom. Peer interaction thus offered participants a way to examine their designs from a classroom user’s perspective.

Together, these patterns suggest a pedagogical rationale for combining the two feedback sources. Participants used AI feedback to consider technical alternatives and validation and used peer feedback to examine classroom realism and learner interpretation. Teacher educators can build on this complementarity by asking PSTs to compare the suggestions, evaluate their assumptions, and explain how selected changes fit together in the revised task.

These observations complement source-comparison work in which AI-generated, peer-generated, or human feedback are compared primarily in writing contexts (Banihashem et al., 2024; Steiss et al., 2024). They also align with broader discussions of generative AI in education that emphasize both affordances and risks (Bearman and Ajjawi, 2023; Kasneci et al., 2023; Yan et al., 2024), while showing that the relevant question for teacher education is how AI feedback is evaluated and integrated with human interpretive resources. The peer-feedback side of this finding is also consistent with research emphasizing the learning value of peer review and feedback use (Liu and Carless, 2006; Nicol et al., 2014; Topping, 1998; van Popta et al., 2017), and situates these benefits in participants’ accounts of classroom realism, student perspective, and mutual communication in modeling-task design.

### 5.4 Sequencing as an ordering context

The condition summaries displayed different engagement trajectories alongside similar composite-quality changes. Mean D1 change was similar across conditions, while the written reasoning classifications differed: the AI-to-peer condition included more strong-consistent engagement, and the peer-to-AI condition included more improving trajectories across rounds. Because assignment occurred at the group level, these are within-course patterns rather than isolated order effects.

One plausible interpretation is that order can foreground different evaluative resources: AI-first feedback may provide an early analytic frame for task components and validation, whereas peer-first feedback may make classroom feasibility and learner interpretation salient before AI introduces additional alternatives. Both explicit rejections occurred in the peer-to-AI condition: one PST rejected a first-round peer suggestion as impractical within a one-week data period, and another rejected a second-round AI suggestion as inconsistent with the task’s central focus, as reflected in their submitted rationales.

For course design, the practical question is how PSTs compare successive feedback and decide what to carry forward. In either order, comparison prompts can ask PSTs to explain how they reconciled, modified, or rejected the second source (Boud and Molloy, 2013; Carless and Winstone, 2023; Hattie and Timperley, 2007). This approach treats sequence as an occasion for evaluative judgment; balanced, counterbalanced studies can test whether particular orders reliably shape engagement or quality.

### 5.5 Implications for feedback design in mathematics teacher education

First, teacher educators can make feedback translation visible through a decision matrix. For each AI or peer suggestion, PSTs record whether they accepted, modified, rejected, or deferred it; identify the targeted task-design dimension; justify the decision; and anticipate trade-offs. This scaffold converts feedback receipt into an explicit exercise in evaluative judgment.

Second, every revision cycle should end with a coherence audit. After changing a prompt, data table, validation step, or decision component, PSTs examine how the change affects openness, modeling demand, contextual authenticity, validation, and opportunities for discussion. This system-level check addresses the translation gap by evaluating the revised task as a coordinated design rather than as a collection of local fixes.

Third, source-differentiated verification should capitalize on the strengths of both feedback forms. PSTs check AI suggestions against grade level, classroom time, student knowledge, data feasibility, and the sustainability meaning of the task. Peer reviewers respond as future teachers and classroom users, testing realism, clarity, and student interpretability. Assessment can then combine final task quality, revision depth, justification of feedback decisions, and reflection on unresolved trade-offs. Together, these practices connect cognitive interpretation, affective trust, and behavioral revision in sustainability-oriented STEM teacher education (Jeong and González-Gómez, 2022).

## 6 Conclusion

This study uses feedback translation as an analytic perspective for tracing how externally generated comments were judged, acted upon, and reflected in task artifacts within an authentic teacher-education course. Across two feedback sequences, PSTs commonly filtered feedback and undertook structural or reconceptual revisions, while rated quality gains appeared in five cases. This divergence illustrates the translation gap as a descriptive pattern: uptake and revision depth were observable aspects of within-activity engagement, whereas the D1 ratings assessed the revised task across context, data, modeling structure, validation, and discussion. Participant records associated AI and peer feedback with differently emphasized resources, bringing source comparison and the integration of selected suggestions into focus.

The study develops this account from one South Korean teacher-education course involving 22 PSTs, unequal AI-to-peer (*n* = 8) and peer-to-AI (*n* = 14) conditions, and a modeling task on school cafeteria food waste. Its contribution lies in tracing how uptake, revision depth, evaluative reasoning, and artifact quality diverged across a complete instructional cycle. As the sources were encountered sequentially within course groups, the account concerns situated feedback use rather than independent source or order effects. Multi-site studies can examine whether these patterns recur across institutions, participant populations, sustainability contexts, and AI systems. Longitudinal research can establish whether the evaluative reasoning documented during the activity persists in later design work and classroom practice. Revision histories, interaction transcripts, interviews, and stimulated recall would help locate the decisions through which PSTs accept, adapt, verify, or reject suggestions. Balanced, counterbalanced comparisons can test the stability of the observed sequence patterns. Design-based studies can examine whether feedback-decision matrices, coherence audits, model-validation workshops, and AI-verification routines help PSTs integrate local revisions into coherent tasks. Together, these approaches would extend this perspective through evidence of recurrence, persistence, and instructional usefulness.

For teacher education, the practical priority is to design feedback cycles that make professional judgment observable and teachable. When PSTs compare sources, justify decisions, verify claims, and audit the coherence of revised artifacts, AI and peer feedback become complementary resources for responsible design. This approach positions feedback as preparation for the work of teaching: interpreting imperfect information, balancing mathematical and pedagogical demands, and constructing tasks that connect rigorous modeling with socially meaningful action.

## Data Availability

The datasets presented in this article are not readily available because they consist of de-identified student coursework, feedback records, revision notes, and reflection data collected in a teacher-education course. Although direct identifiers were removed, the data may contain contextual information that could increase the risk of participant re-identification. Access is therefore limited and may be considered only upon reasonable request to the corresponding author, subject to institutional ethics approval and applicable data-protection requirements. No participant-identifiable data will be shared. Requests to access the datasets should be directed to the corresponding author (soh@hongik.ac.kr).

## Footnotes

1
https://chatgpt.com/g/g-69d43a997e4c8191979081d61b7eb882-suhagjeog-modelring-hwaldong
